# Differences in the flexion and extension phases during kneeling investigated by kinematic and contact point analyses: a cross-sectional study

**DOI:** 10.1186/s13018-022-03080-x

**Published:** 2022-03-28

**Authors:** Yusuke Nakazoe, Akihiko Yonekura, Hiroyuki Takita, Takeshi Miyaji, Narihiro Okazaki, Ko Chiba, Kenichi Kidera, Takashi Miyamoto, Masato Tomita, Kazuyoshi Gamada, Makoto Osaki

**Affiliations:** 1 Department of Orthopaedic Surgery, Nagasaki University Graduate School of Biomedical Sciences, 1-7-1 Sakamoto, Nagasaki, 852-8501 Japan; 2 Medical Engineering and Technology, Graduate School of Medical Technology and Health Welfare Sciences, Hiroshima International University, 553-36 Kurosegakuendai, Hiroshima, 739-2695 Japan

**Keywords:** Kneeling, Kinematics, Deep knee flexion, Healthy knee, 2D/3D registration technique

## Abstract

**Background:**

Kneeling is necessary for certain religious and ceremonial occasions, crouching work, and gardening, which many people take part in worldwide. However, there have been few reports about kneeling activities. The purpose of this study was to clarify the kinematics of kneeling.

**Methods:**

The subjects were 15 healthy young males. Kneeling activity was analysed within a knee flexion angle from 100° to maximum flexion (maxflex, mean ± SD = 161.3 ± 3.2°). The kinematic and contact point (CP) analyses were performed using a 2D/3D registration method, in which a 3D bone model created from computed tomography images was matched to knee lateral fluoroscopic images and analysed on a personal computer.

**Results:**

In the kinematic analysis, the femur translated 37.5 mm posteriorly and rotated 19.8° externally relative to the tibia during the knee flexion phase. During the knee extension phase, the femur translated 36.4 mm anteriorly, which was almost the same amount as in the knee flexion phase. However, the femur rotated only 7.4° internally during the knee extension phase. In the CP analysis, the amount of anterior translation of the CP in the knee extension phase was greater in the medial CP and smaller in the lateral CP than that of posterior translation in the knee flexion phase.

**Conclusions:**

In kneeling, there was a difference in the rotational kinematics between the flexion phase and the extension phase. The kinematic difference between the flexion and extension phases may have some effect on the meniscus and articular cartilage.

## Background

Kneeling is a movement that flexes the knee joint deeply with the tibial tubercle on the ground. It is one of the deep knee flexion activities, like squatting or lunging. Kneeling is required for religious and ceremonial occasions in Asia. The posture that holds the deep knee flexion position is called “Seiza” in Japan. The prevalence of knee osteoarthritis (OA) was reported to be relatively high, at 60% or more, in Asia, compared with 30–37.4% in Europe and the USA [1–4]. One of the reasons is that Asians have a relatively high frequency of kneeling activities in religion and daily life, which may be related to the occurrence of OA. Kneeling is also often done in activities such as crouching work and gardening in western countries. The relationship between the onset of knee joint diseases (such as OA and meniscal injury) and the occupations requiring frequent deep knee flexion has been reported in recent years [5–8]. During kneeling activity, a series of knee joint kinematics of flexion and extension may be repeated many times, which may have an effect on intra-articular components such as the menisci and cartilage. Therefore, it is important to clarify the characteristic kinematics of kneeling.

Deep knee flexion analysis has been reported since the late 1990s in static and cadaver knee studies [9–13]. There are many reports of dynamic studies of knee kinematics after total knee arthroplasty, and several of deep knee flexion and kneeling situations have already been reported [14–19]. Subsequently, there have appeared some reports of the kinematics of squat and lunge activities in dynamic studies of healthy knees [20–23]. However, there have been only a few reports of kneeling of healthy knees [24–27]. According to these reports, the femur translates posteriorly and rotates externally during deep knee flexion. On the other hand, contact point (CP) analysis has been used as a method for analysing the movements of the medial and lateral femorotibial CPs separately [22, 24, 28]. It has been reported that there were significant differences in the lateral CP and no differences in the medial CP in the flexion phase of kneeling [24]. However, no reports have investigated the knee extension phase. Kneeling is different from squatting and lunging in that the femoral condyles move on the tibial plateau that is almost perpendicular to the ground. A gravitational force on the femur that slides down on the tibial plateau always affects kneeling. The anterior translation of the femur on the tibial plateau with this gravitational force in the knee extension phase may be different from the posterior translation of the femur against this gravitational force in the knee flexion phase.

A previous study reported that there is no difference in the kinematics of kneeling activity between the extension phase and the flexion phase [26]. However, the subjects in that study varied in sex and were relatively old. We planned to clarify the distinctive kinematics of kneeling by analysing only knees in young males with no apparent trauma history or OA. In addition, we planned to investigate the movement of medial and lateral CPs not only in the flexion phase, but also in the extension phase. To clarify the kinematics and CPs that have not been elucidated in the previous studies of kneeling activity, we hypothesized the following:

1. The kinematics of femoral translation and/or rotation in the extension phase are different from those in the flexion phase.
2. The movements of the medial and lateral CPs differ between the flexion phase and the extension phase.

## Methods

### Subjects

This study was conducted as part of a cross-sectional study. According to the protocol approved by the hospital ethics committee (approval number: 08070298-2), subjects were selected from hospital patients. The inclusion criteria were: (1) males over 20 years of age; (2) those with no history of trauma or surgery on the subject knee; (3) those with full knee extension and knee flexion > 145°; (4) X-ray examination showing no OA; and (5) those who gave informed consent. Females were excluded from the present study to avoid radiation exposure because of the possibility of pregnancy. Ultimately, 15 males (15 knees) with an average age of 28.2 ± 6.9 years, an average height of 169.9 ± 6.5 cm, an average weight of 70.4 ± 11.9 kg, and an average body mass index of 25.3 ± 5.1 kg/m^2^ were included.

### Kneeling action and lateral fluoroscopic imaging

**Fig. 1:**
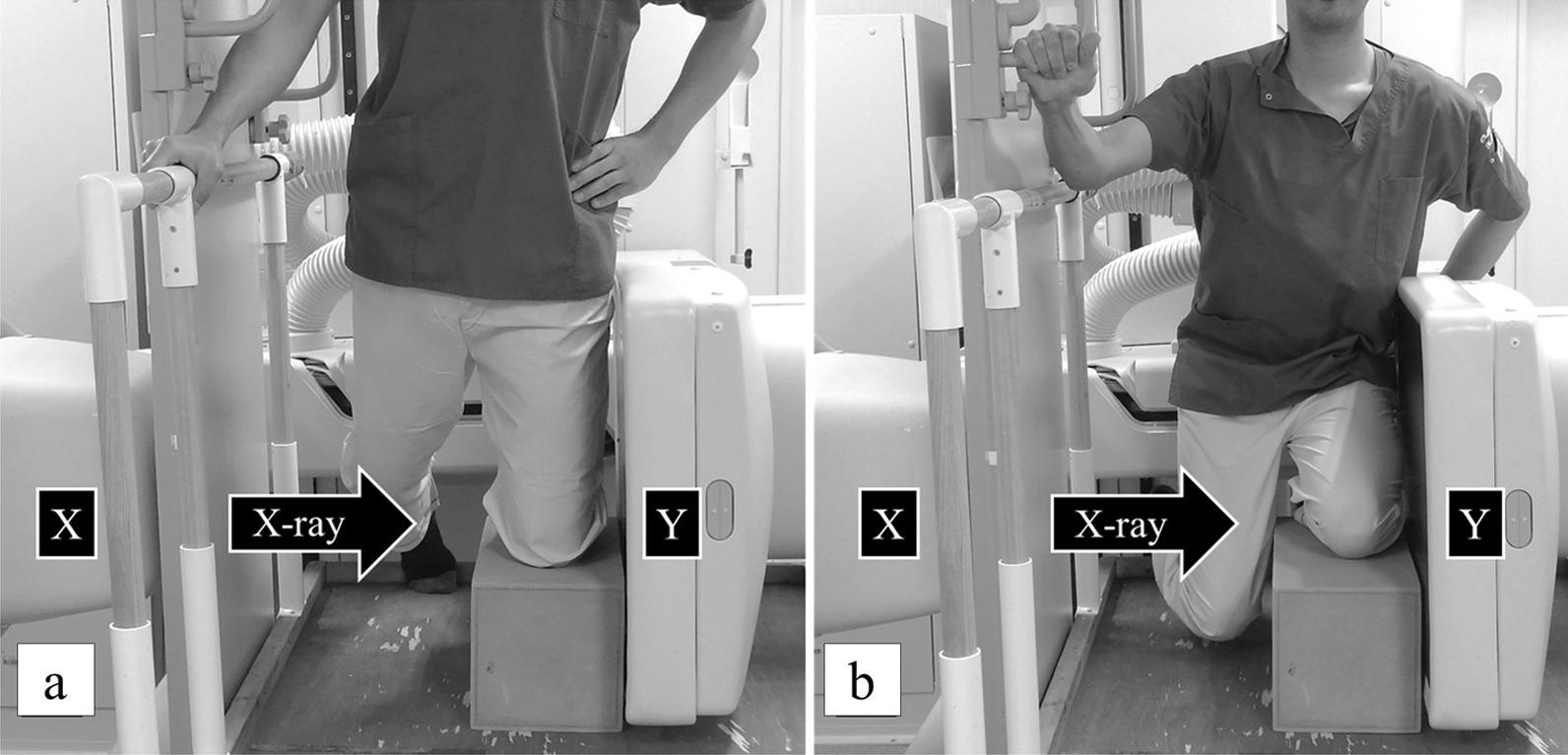
Kneeling action and lateral fluoroscopic imaging. **a** Represents the starting posture when the subject is kneeling with a knee flexion angle of 90°. **b** Represents the posture when the subject flexes the knee to the maximum flexed angle from (**a**). X-rays are emitted from the radiation source (X) toward the flat panel (Y), and a series of actions are recorded

As for the starting posture, the frontal part of the lower leg was placed on a radiolucent box (Fig. 1a). The starting knee posture was set to a knee flexion angle of 90° measured by the goniometer. The hip joint was in the intermediate flexion/extension position, and the ankle was not fixed and was able to move freely. The contralateral leg was placed backwards so that it did not overlap the examined knee on the fluoroscopic image. The subjects were tested while holding onto a handrail for safety and in order to apply half of their body weight to the examined knee. To minimize the exposure dose, the subjects wore a radiation protector on the hips during fluoroscopy.

The subject was instructed to perform the reciprocating movement over about 5 s so as not to sway from side to side during image capture. The subject practiced several times until smooth kneeling action was achieved. First, the images of the flexion phase were captured. The subject then started to kneel down from a knee flexion angle of 90° toward the maximum flexed knee position until his buttock touched the heel (Fig. 1b). Then, a series of actions returning from the maximum flexed knee position to a knee flexion angle of 90° were performed as the extension phase (returned to the same posture as Fig. 1a). The actual kneeling motion took 6–8 s, similar to previous reports [26].

The fluoroscope used in this study was a square, 17-inch, flat panel screen (C-vision Safire, Shimadzu Corp., Kyoto, Japan). The imaging frame rate was 5 Hz, and the image size was 1024 × 1024 pixels. A series of actions were recorded as the kinematic data. Static images were extracted from the kinematic data. The flexion phase and the extension phase were analysed separately.

### 3D bone model creation and coordinate system embedding

The 3D bone models of the femur and tibia were created from CT images (SOMATOM Definition, Siemens AG, Erlangen, Germany). In all cases, CT scans were performed with 0.5-mm-thick slices, approximately 150 mm proximal and distal from the knee joint line. 3D-Doctor (Able Software Corp., Lexington, MA, USA) was used to surround the exterior cortical bone edges of each axial image one by one. 3D bone models were created based on these images.

**Fig. 2:**
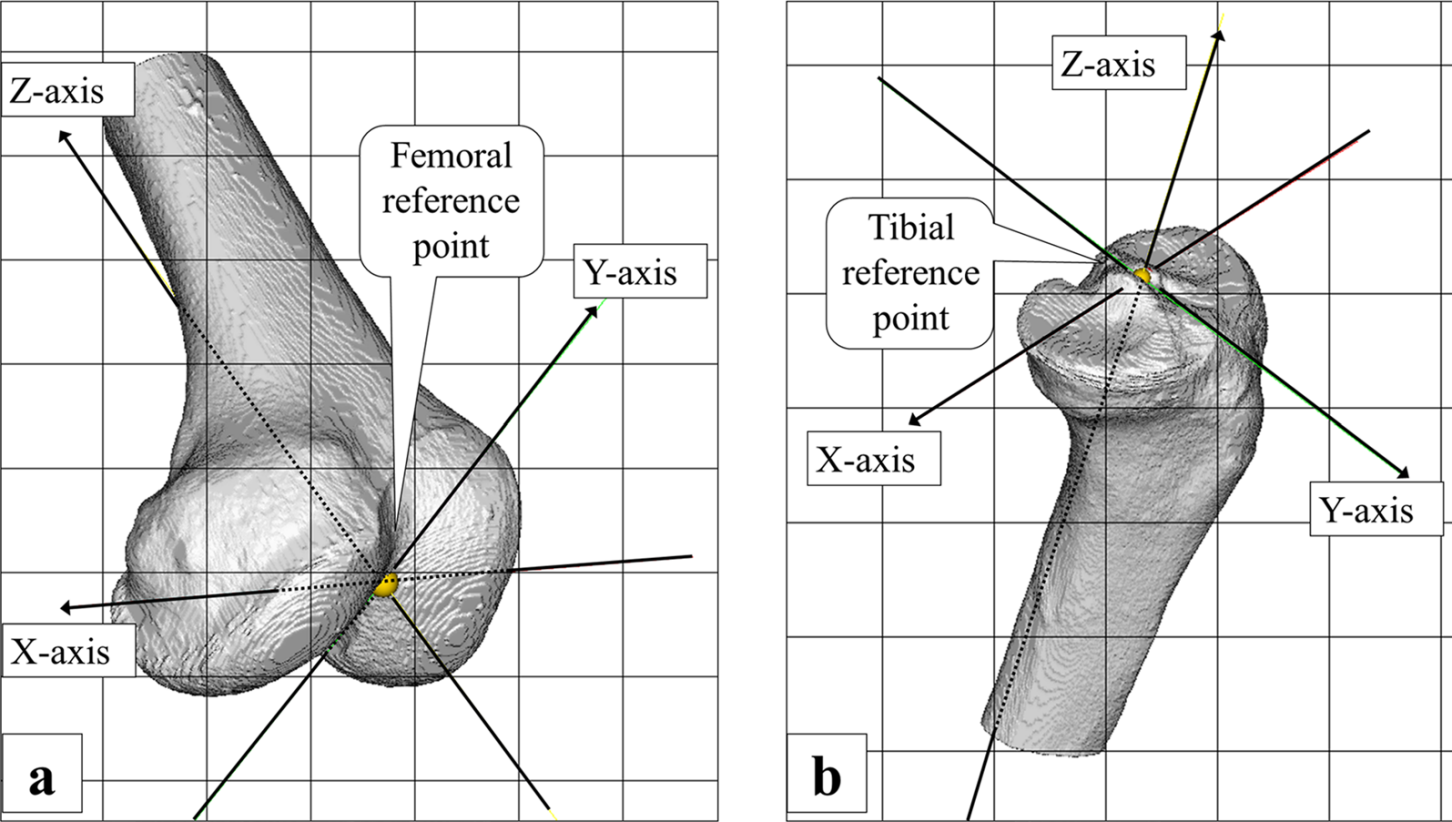
Femoral and tibial coordinate systems. **a** Shows the femoral coordinate system. The femoral reference point is located at the most distal point on the mid-plane of the intercondylar notch. The Z-axis points proximally and passes through the centre of the femoral head. The X-axis passes the femoral reference point and is parallel to the line connecting the most posterior points of the two femoral condyles. The Y-axis is mutually perpendicular to the X- and Z-axes. **b** Shows the tibial coordinate system. The tibial reference point is located at the centre of the apex of the medial–lateral intercondylar ridge and was the lowest point on the bone surface. The Z-axis points proximally and passes from the centre of the ankle joint and through the tibial reference point. The X-axis passes the tibial reference point and is parallel to the line connecting the midpoints of the medial and lateral tibial condyles. The Y-axis is mutually perpendicular to the X- and Z-axes

The bone coordinate system was set using 3D-Aligner (GLAB Corp., Higashi-Hiroshima, Japan). A coordinate system was incorporated into each bone model of the femur and tibia (Fig. 2). The femoral and tibial coordinate system was based on the definition of Grood and Suntay [29]. The femoral reference point was located at the most distal point on the mid-plane of the intercondylar notch. The Z-axis pointed proximally and passed through the centre of the femoral head. The X-axis passed the femoral reference point and was parallel to the line connecting the most posterior points of the two femoral condyles. The Y-axis was mutually perpendicular to the X- and Z-axes. The tibial reference point was located at the centre of the apex of the medial–lateral intercondylar ridge and was the lowest point on the bone surface. The Z-axis pointed proximally and passed from the centre of the ankle joint and through the tibial reference point. The X-axis passed the tibial reference point and was parallel to the line connecting the midpoints of the medial and lateral tibial condyles. The Y-axis was mutually perpendicular to the X- and Z-axes.

### Kinematic and contact point analyses

Knee joint kinematics and contact points were analysed by the 2D/3D registration method using X-ray lateral fluoroscopic images and CT images of the knee joint developed by Banks et al. [30]. Previous reports have shown that this method is accurate and has very few errors [31]. Moro-oka et al. reported that the accuracy of this technique was 0.53 mm for in-plane translation, 1.6 mm for out-of-plane translation, and 0.54° for rotation [32]. Komistek et al. reported that accuracy was 0.45 mm for in-plane translation, 4.0 mm for out-of-plane translation, and 0.66° for rotation [33]. We previously used this method to investigate squatting activity [34, 35].

**Fig. 3:**
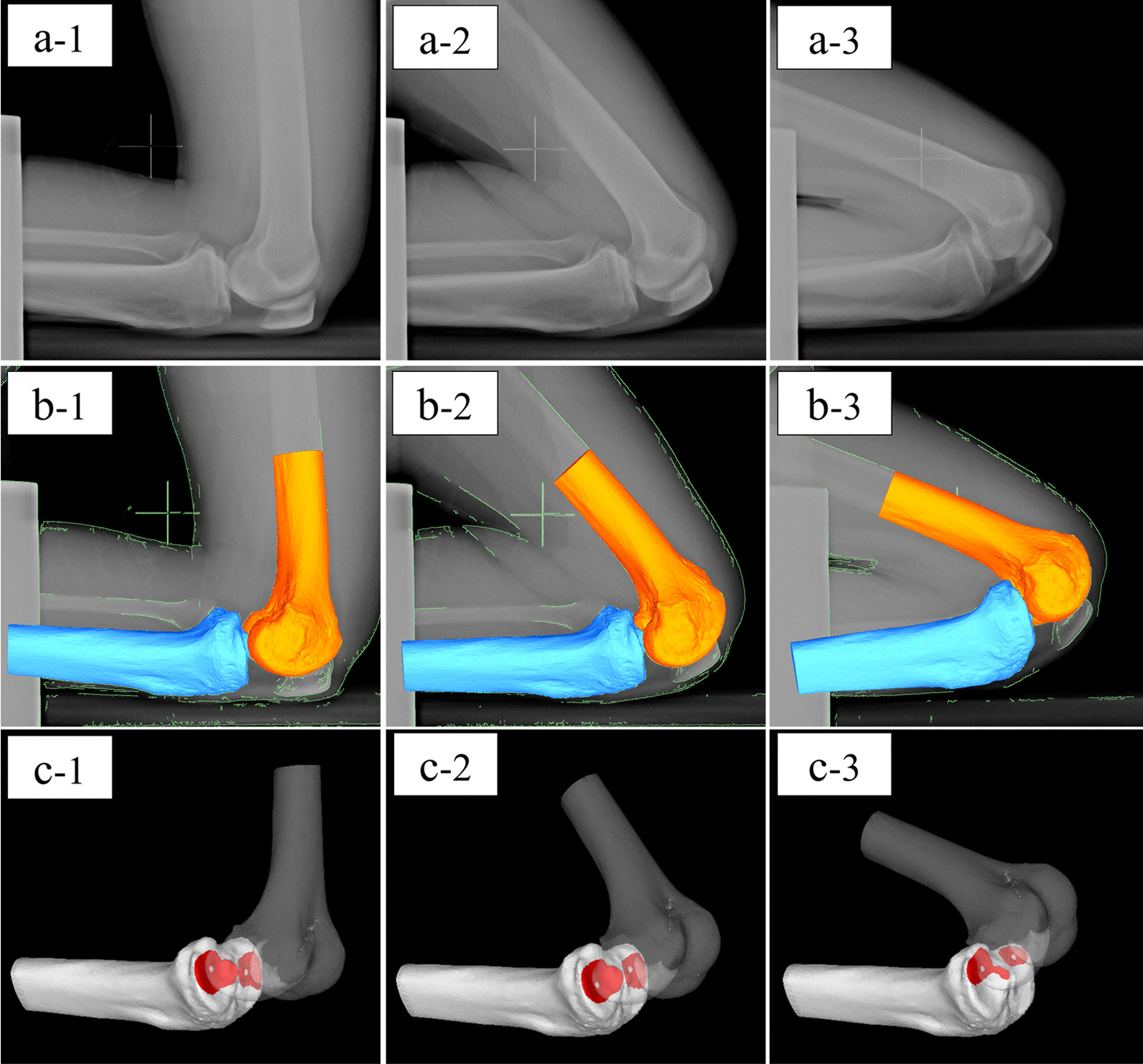
Kinematic and contact point analysis of the flexion phase. **a** Presents the lateral fluoroscopic images. **b** Presents the bone models adjusted to match the lateral fluoroscopic images. The red area of **c** is the contact area, and the white point is the contact point that is the geometric centre of the contact area. The number 1 represents the starting position of kneeling activity, the number 2 represents the middle of flexion of the knee, and the number 3 represents the maximum flexion of the knee

The 3D bone models embedded with the coordinate system were projected onto the distortion-corrected fluoroscopic images. The silhouettes of the bone models were iteratively adjusted to match the silhouettes of the bones on the fluoroscopic image with the custom Joint Track program (sourceforge.net/projects/jointtrack) (Fig. 3). Then, six degrees-of-freedom joint kinematics were computed using commercial software (3DJointManager, GLAB Corp.).

Kinematic analysis was assessed by the movement of the femur relative to the tibia. The six degrees of freedom that were measured were three rotations (flexion, varus-valgus (VV), and internal–external (IE)) and three translations (anterior–posterior (AP), medial–lateral (ML), and inferior-superior (IS)) [27, 29]. The B-spline curve approximation was selected, as in our previous reports, to reproduce the smooth movement of the knee joints [34, 35]. The kinematics were analysed in 5° increments of the knee flexion angle after B-spline curve approximation. In all data processing, only interpolation was performed; extrapolation was not performed.

In addition, CP analysis was performed. The contact area was analysed using the same commercial software (3DJointManager, GLAB Corp.) used in the kinematic analysis. The motions of medial and lateral femoral condyles have been evaluated by the medial and lateral CPs, respectively [22, 28]. The bone surfaces of the femur and tibia are composed of a collection of polygons. The distance between polygons composed of the bone surfaces of the femur and tibia was calculated as the proximity distance. An area within a certain proximity distance was defined as the contact area. The minimum proximity distance at which the contact area appeared varied depending on the subject, with a minimum of 5.5 mm and a maximum of 7.0 mm. The geometric centre, which is the centre of gravity of the contact area, was defined as the CP. The CP was represented by the coordinate position on the tibial X–Y plane. Each medial and lateral CP was digitized separately, and a series of movements of the CPs were evaluated for each knee flexion angle.

### X-ray exposure dose

In the present study, the subjects’ X-ray exposure doses were confirmed to be 22 mSv with fluoroscopy and 8 mSv with CT. Only one cycle of kneeling action was allowed on fluoroscopic examination to minimize the exposure dose [35].

### Statistical analyses

SPSS version 22 (SPSS Inc., Chicago, IL, USA) was used for statistical analysis. A paired *t*-test was used for comparison between the flexion phase and the extension phase with the knee flexion angle as the independent variable. P < 0.05 was considered significant.

The difference between the flexion phase and the extension phase at each flexion angle of each case was calculated. Using a mixed model for repeated measurements with the numerical value of the difference as the objective variable, whether there was an overall difference between the flexion phase and the extension phase was evaluated.

## Results

### Kinematic analysis

**Fig. 4:**
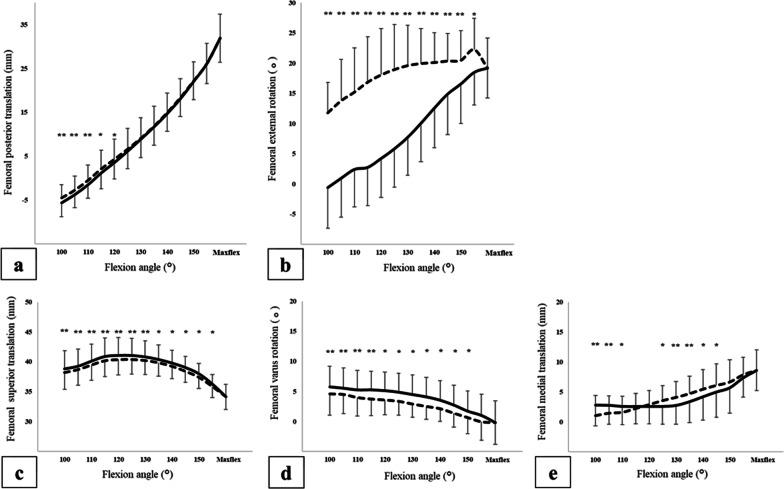
Kinematic data of the femur relative to the tibia. **a** Shows the femoral anterior–posterior translation (mm), plus indicates posterior translation, and minus indicates anterior translation. **b** Shows femoral internal–external rotation (°), plus indicates external rotation, and minus indicates internal rotation. **c** Shows the femoral inferior-superior translation (mm), plus indicates superior translation, and minus indicates inferior translation. **d** Shows femoral varus-valgus rotation (°), plus indicates varus rotation, and minus indicates valgus rotation. **e** Shows the femoral medial–lateral translation (mm), plus indicates medial translation, and minus indicates lateral translation. The horizontal axis shows the knee flexion angle. The solid line represents the flexion phase, and the dashed line represents the extension phase. Paired *t*-test; *P < 0.05, **P < 0.01

The femur translated posteriorly in the flexion phase and anteriorly in the extension phase relative to the tibia on the kinematic analysis (Fig. 4a). The average amount of femoral posterior translation during the knee flexion phase from 100° to maximum flexion (maxflex, mean ± SD = 161.3 ± 3.2°) was 37.5 mm, and it was -36.4 mm during the extension phase from maxflex to 100° (Table 1). Comparing the femoral posterior translation for each knee flexion angle, there were significant differences between the two phases from a knee flexion angle of 100° to 120°. The estimated overall difference for femoral AP translation between the flexion and extension phases was 0.48 mm (95% confidence interval (CI) 0.08 to 0.87 mm).

**Table 1:** Posterior translation and external rotation of the femur relative to the tibia

Knee flexion angle (°)	*n* (knees)	Posterior translation (mm)				External rotation (°)			
		Flexion phase	(SD)	Extension phase		Flexion phase	(SD)	Extension phase	(SD)
100	11	− 5.6	(3.2)	− 4.5**	(3.0)	− 0.6	(5.0)	11.8**	(6.7)
105	13	− 3.7	(3.1)	− 2.7**	(3.2)	1.0	(6.8)	13.8**	(6.5)
110	14	− 1.5	(3.1)	− 0.5**	(3.5)	2.4	(7.3)	15.2**	(6.2)
115	15	1.2	(3.7)	2.1*	(4.3)	2.8	(7.5)	16.9**	(6.4)
120	15	3.6	(3.8)	4.3*	(4.6)	4.2	(7.7)	18.1**	(6.4)
125	15	6.2	(4.1)	6.6	(4.7)	5.8	(7.4)	19.0**	(6.3)
130	15	8.9	(4.2)	9.1	(4.6)	7.7	(6.7)	19.6**	(6.3)
135	15	11.7	(4.2)	11.9	(4.5)	10.1	(5.8)	20.0**	(6.4)
140	15	14.8	(4.1)	14.9	(4.4)	12.5	(4.9)	20.1**	(6.6)
145	15	18.2	(4.1)	18.4	(4.3)	14.9	(4.6)	20.4**	(6.7)
150	15	22.1	(4.2)	22.2	(4.3)	16.6	(4.9)	20.5**	(6.5)
155	12	26.2	(4.7)	26.1	(4.6)	18.5	(5.2)	22.3*	(5.4)
Maxflex	12	31.9	(5.5)	31.9	(5.5)	19.2	(5.0)	19.2	(5.0)
Range		37.5		36.4		19.8		7.4	

Paired *t*-test; *P < 0.05, **P < 0.01

SD, standard deviation; maxflex, maximum flexion

The femur rotated externally with knee flexion and internally with knee extension relative to the tibia on the kinematic analysis (Fig. 4b). The average amount of femoral external rotation during the knee flexion phase from 100° to maxflex was 19.8°, and it was − 7.4° during the extension phase from maxflex to 100° (Table 1). Comparing femoral external rotation for each knee flexion angle, there was a significant difference between the two phases from a knee flexion angle of 100° to 155°. The estimated overall difference for femoral IE rotation between the flexion and extension phases was − 9.86° (95% CI − 13.75° to − 5.97°).

The femur translated inferiorly in the flexion phase and superiorly in the extension phase relative to the tibia on the kinematic analysis (Fig. 4c). The average amount of femoral inferior translation during the knee flexion phase from 100° to maxflex was 4.7 mm, and it was -4.1 mm during the extension phase from maxflex to 100° (Table 2). Comparing the femoral superior translation for each knee flexion angle, there were significant differences between the two phases from a knee flexion angle of 100° to 150°. The estimated overall difference for femoral IS translation between the flexion and extension phases was 0.55 mm (95% CI 0.28 to 0.81 mm).

**Table 2:** Medial–lateral translation, varus-valgus rotation, and inferior-superior translation of the femur relative to the tibia

Knee flexion angle (°)	*n* (knees)	Superior translation (mm)				Varus rotation (°)				Medial translation (mm)			
		Flexion phase	(SD)	Extension phase	(SD)	Flexion phase	(SD)	Extension phase	(SD)	Flexion phase	(SD)	Extension phase	(SD)
100	11	38.8	(3.1)	38.2**	(2.8)	5.7	(3.5)	4.6**	(3.5)	2.8	(3.5)	1.0**	(3.3)
105	13	39.2	(2.9)	38.7**	(2.7)	5.5	(3.4)	4.5**	(3.2)	2.8	(3.2)	1.5**	(2.9)
110	14	40.1	(2.9)	39.5**	(2.6)	5.2	(3.3)	4.0**	(3.1)	2.6	(3.0)	1.6*	(2.6)
115	15	40.9	(3.1)	39.5**	(2.7)	5.2	(3.1)	3.7**	(2.8)	2.6	(2.9)	2.2	(2.5)
120	15	41.0	(3.0)	40.4**	(2.6)	5.1	(3.1)	3.6*	(2.5)	2.6	(2.9)	2.9	(2.4)
125	15	41.0	(2.9)	40.4**	(2.5)	4.9	(3.2)	3.3*	(2.3)	2.6	(3.0)	3.6*	(2.5)
130	15	40.8	(2.7)	40.2*	(2.4)	4.5	(3.3)	2.9*	(2.2)	2.8	(3.1)	4.1**	(2.6)
135	15	10.4	(2.5)	39.9*	(2.3)	4.1	(3.2)	2.5*	(2.2)	3.4	(3.5)	4.7**	(2.9)
140	15	39.8	(2.2)	39.3*	(2.1)	3.5	(3.3)	2.1*	(2.1)	4.2	(3.9)	5.5*	(3.3)
145	15	39.0	(1.9)	38.4*	(1.9)	2.7	(3.3)	1.4*	(2.3)	5.0	(4.2)	6.1*	(3.6)
150	15	38.0	(1.7)	37.4*	(1.9)	1.7	(3.3)	0.6*	(2.6)	5.7	(4.3)	6.7	(3.7)
155	12	36.2	(1.6)	35.8	(1.9)	1.0	(3.5)	− 0.1*	(3.1)	7.4	(3.2)	7.9	(2.9)
Maxflex	12	34.1	(2.1)	34.1	(2.1)	− 0.2	(3.6)	− 0.2	(3.6)	8.6	(3.4)	8.6	(3.4)
Range		4.7		4.1		5.9		4.8		5.8		7.6	

Paired *t*-test; *P < 0.05, **P < 0.01

SD, standard deviation; maxflex, maximum flexion

The femur rotated into valgus in the flexion phase and into varus in the extension phase relative to the tibia on the kinematic analysis (Fig. 4d). The average amount of femoral varus rotation during the knee flexion phase from 100° to maxflex was -5.9°, and it was 4.8° during the extension phase from maxflex to 100° (Table 2). Comparing the femoral varus rotation for each knee flexion angle, there were significant differences between the two phases from a knee flexion angle of 100° to 155°. The estimated overall difference for femoral VV rotation between the flexion and extension phases was 1.34° (95% CI 0.51° to 2.1°).

The femur translated medially in the flexion phase and laterally in the extension phase relative to the tibia on the kinematic analysis (Fig. 4e). The average amount of femoral medial translation during the knee flexion phase from 100° to maxflex was 5.8 mm, and it was -7.6 mm during the extension phase from maxflex to 100° (Table 2). Comparing the femoral medial translation for each knee flexion angle, there were significant differences between the two phases from a knee flexion angle of 100° to 110° and from 125° to 145°. The estimated overall difference for femoral ML translation between the flexion and extension phases was 0.27 mm (95% CI -1.06 to 0.52 mm).

In summary, the amounts of AP translation, IS translation, VV rotation, and ML translation were almost the same in the two phases, but the amount of femoral IE rotation was less in the extension phase than in the flexion phase on the kinematic analysis.

### Contact point analysis

**Fig. 5:**
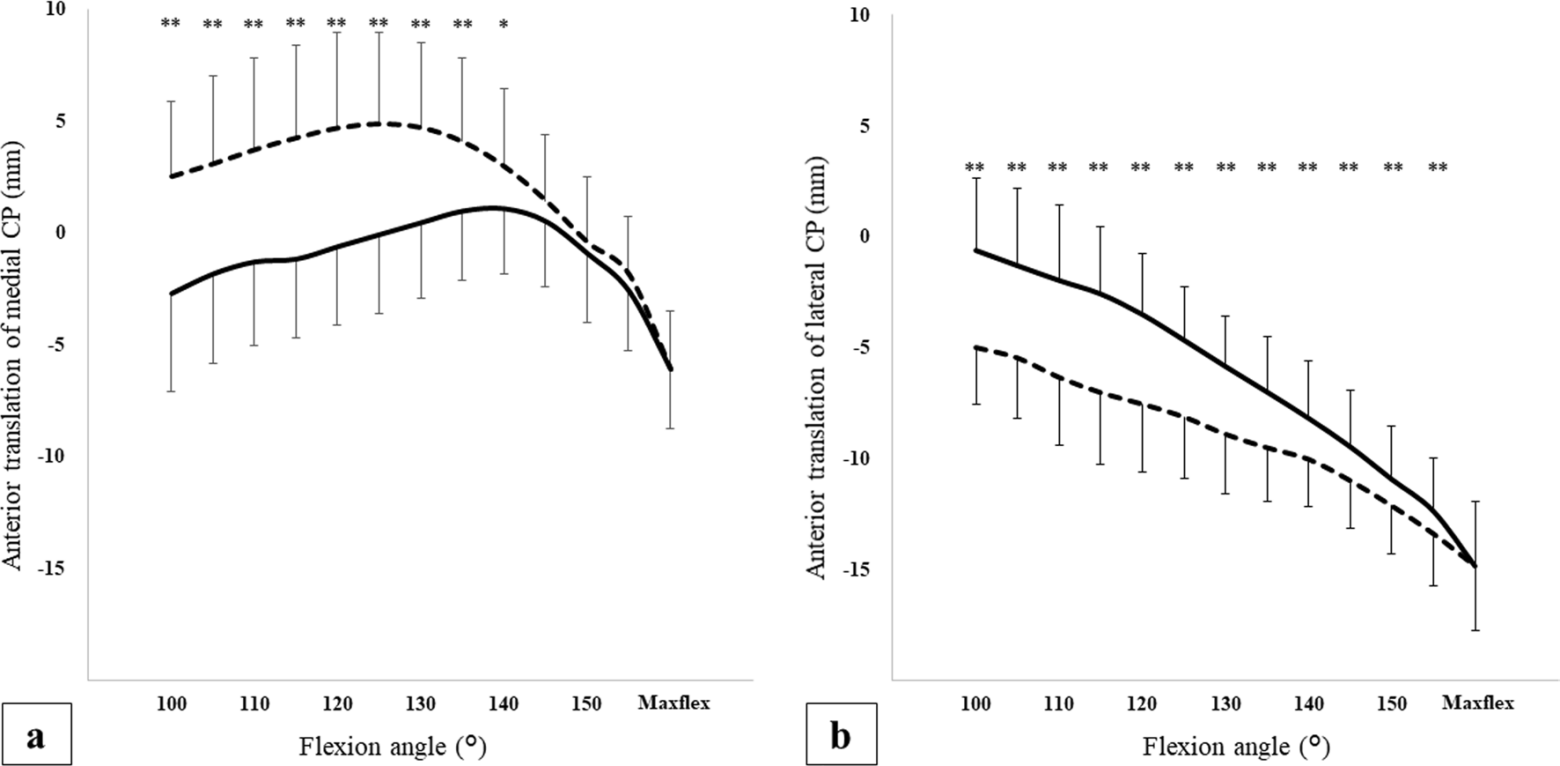
Antero-posterior translation of medial and lateral contact points. **a** Shows anterior–posterior (AP) translation (mm) of the medial contact point (CP). **b** Shows AP translation (mm) of the lateral CP. Plus indicates anterior translation of the CP, and minus indicates posterior translation of the CP. The horizontal axis shows the knee flexion angle. The solid line represents the flexion phase, and the dashed line represents the extension phase. Paired *t*-test; *P < 0.05, **P < 0.01

The medial CP translated 3.4 mm posteriorly with a knee flexion angle from 100° to maxflex in the flexion phase: 3.8 mm anteriorly from 100° to 140°, and 7.2 mm posteriorly from 140° to maxflex (Fig. 5a, Table 3). In the extension phase, the medial CP translated 8.6 mm anteriorly with a knee flexion angle from maxflex to 100°, 11.0 mm anteriorly from maxflex to 125°, and 2.4 mm posteriorly from 125° to 100°. There was a significant difference between the two phases with a knee flexion angle from 100° to 140°. The estimated overall difference for the medial CP translation between the flexion and extension phases was -3.41 mm (95% CI -4.88 to -1.93 mm). That is, the medial CP was located more anteriorly in the extension phase than in the flexion phase.

**Table 3:** Anterior translation of the medial CP

Knee flexion angle (°)	*n* (knees)	Flexion phase			Extension phase			*P* value
		Anterior translation (mm)	(SD)	Difference from 140°	Anterior translation (mm)	(SD)	Difference from 125°	
100	11	− 2.7	(3.4)	− 3.8	2.5	(4.4)	− 2.4	< 0.001**
105	13	− 1.8	(3.9)	− 2.9	3.1	(4.0)	− 1.8	< 0.001**
110	14	− 1.3	(4.1)	− 2.4	3.7	(3.7)	− 1.2	< 0.001**
115	15	− 1.2	(4.1)	− 2.3	4.3	(3.5)	− 0.6	< 0.001**
120	15	− 0.6	(4.3)	− 1.7	4.7	(3.5)	− 0.2	< 0.001**
125	15	− 0.1	(4.1)	− 1.2	4.9	(3.5)	0	< 0.001**
130	15	0.5	(3.8)	− 0.6	4.7	(3.4)	− 0.2	< 0.001**
135	15	1.0	(3.7)	− 0.1	4.1	(3.1)	− 0.8	< 0.001**
140	15	1.1	(3.5)	0	3.0	(2.9)	− 1.9	0.031*
145	15	0.5	(3.0)	− 1.6	1.5	(2.9)	− 3.4	0.23
150	15	− 0.9	(2.9)	− 2.0	− 0.4	(3.1)	− 5.3	0.44
155	12	− 2.6	(2.6)	− 3.7	− 1.9	(2.7)	− 6.8	0.28
Maxflex	12	− 6.1	(2.6)	− 7.2	− 6.1	(2.6)	− 11.0	–
Range		3.4			8.6			

Paired *t*-test; *P < 0.05, **P < 0.01

CP: contact point; SD: standard deviation; maxflex: maximum flexion

The lateral CP translated continuously 14.2 mm posteriorly with a knee flexion angle from 100° to maxflex in the flexion phase (Fig. 5b, Table 4). Similarly, the lateral CP translated continuously 9.8 mm anteriorly with a knee flexion angle from maxflex to 100° in the extension phase. There was a significant difference between the two phases with a knee flexion angle from 100° to 155°. The estimated overall difference for the lateral CP translation between the flexion and extension phases was 2.92 mm (95% CI 1.82 to 4.01 mm). That is, the lateral CP was located more posteriorly in the extension phase than in the flexion phase.

**Table 4:** Anterior translation of the lateral CP

Knee flexion angle (°)	*n* (knees)	Flexion phase			Extension phase			*P* value
		Anterior translation (mm)	(SD)	Difference from 100°	Anterior translation (mm)	(SD)	Difference from maxflex	
100	11	− 0.6	(3.3)	0	− 5.0	(2.6)		< 0.001**
105	13	− 1.3	(3.5)	− 0.7	− 5.4	(2.7)	9.4	< 0.001**
110	14	− 2.0	(3.4)	− 1.4	− 6.3	(3.0)	8.5	< 0.001**
115	15	− 2.6	(3.0)	− 2.0	− 7.0	(3.2)	7.8	< 0.001**
120	15	− 3.5	(2.7)	− 2.9	− 7.5	(3.1)	7.3	< 0.001**
125	15	− 4.6	(2.4)	− 4.0	− 8.1	(2.8)	6.7	< 0.001**
130	15	− 5.8	(2.3)	− 5.2	− 8.9	(2.7)	5.9	< 0.001**
135	15	− 7.0	(2.5)	− 6.4	− 9.5	(2.4)	5.3	< 0.001**
140	15	− 8.2	(2.6)	− 7.6	− 10.0	(2.1)	4.8	0.003**
145	15	− 9.4	(2.5)	− 8.8	− 11.0	(2.1)	3.8	0.002**
150	15	− 10.9	(2.4)	− 10.3	− 12.1	(2.2)	2.7	0.003**
155	12	− 12.3	(2.4)	− 11.7	− 13.4	(2.4)	1.4	0.004**
Maxflex	12	− 14.8	(2.9)	− 14.2	− 14.8	(2.9)	0	–
Range		14.2			9.8			

Paired *t*-test; *P < 0.05, **P < 0.01

CP, contact point; SD, standard deviation; maxflex, maximum flexion

## Discussion

The present results showed that the amount of femoral IE rotation was smaller in the extension phase than in the flexion phase, which has not been reported previously. Furthermore, large anterior translation of the medial femoral condyle in the extension phase compared with in the flexion phase was observed, which is also a new finding.

The relationship between kneeling activity with meniscus injury and knee OA has been shown epidemiologically. In a cohort study, Jensen et al. reported that kneeling workers had an odds ratio of 2.82 (95% CI 1.25 to 6.36) for medial meniscus injury, and workers repetitively kneeling for more than 30 years had an odds ratio of 4.82 (95% CI 1.38 to 17.0) for femorotibial OA, compared with non-kneeling workers [7]. On the other hand, Nagura et al. analysed the moment and force applied to the knee joint during squatting and kneeling using a motion capture system with a ground reaction force plate [36–38]. They reported that the contact pressure of the knee joint was very high in the deep knee flexion position. Nakagawa et al. observed the meniscus by open MRI at various knee flexion angles and reported that the medial meniscus was sandwiched between the femoral condyle and the posterior part of the tibial condyle at the maximum knee flexion position [12]. Furthermore, it was reported that the posterior horn of the medial meniscus had the least amount of movement, with a potential risk of meniscus damage [39, 40]. In addition to these intra-articular states, the repetition of different IE rotational kinematics and CP translation in the flexion and the extension phases in activities of daily living and work might be involved in medial meniscus injury and development of OA.

Although there are several reports of the kinematic analysis of kneeling, this study showed for the first time that there was a difference in the IE rotational kinematics between the flexion phase and the extension phase. Regarding kneeling in the flexion phase, the previous reports showed that the femur rotated externally and translated posteriorly with flexion [24–27]. In addition, it has also been reported that there was little IS translation, ML translation, and VV rotation of the femur [26, 27]. Thus, the present result of the kinematic analysis of kneeling activity in the flexion phase was in the same direction of IE rotation and AP translation of the femur seen in previous reports. In addition, the amounts of IS translation, ML translation, and VV rotation of the femur were as small as in previous reports.

To the best of our knowledge, only two reports have shown the kinematic data both in the flexion and in the extension phases in kneeling using the 2D/3D registration method. Scarvell et al. analysed kneeling in both the flexion and extension phases, and they reported that kneeling required femoral posterior translation and external rotation [26]. Galvin et al. analysed kneeling in four 20-year age groups and reported that there was no relationship between aging and the inability to kneel, except in the over 80-year age group [27]. However, they did not mention the difference in the amount of IE rotation between the flexion phase and the extension phase. In the present results, the amount of IE rotation was smaller in the extension phase than in the flexion phase. The reason for this difference might be related to the ethnic backgrounds and the age and sex distributions of the subjects. Whereas the subjects of the previous reports were Caucasians, the subjects of the present study were Asians. Furthermore, the present subjects were only males with an average age of 28.2 years, whereas the subjects in the study by Scarvell et al. were 13 males and 12 females with an average age of 62 years, and subjects in the study by Galvin et al. were 30 males and 36 females in various age groups. Although the number of cases in the present study was less than in their studies, the difference between the flexion phase and the extension phase in the present study, which has not been reported previously, may be a result of the potential tolerance and flexibility of a healthy knee of a relatively young generation in Asians.

To the best of our knowledge, only two reports have analysed the translation of the medial and lateral femoral condyles individually in the flexion phase of kneeling using the 2D/3D registration method [24, 25]. These reports have shown that the AP translation of the medial CP was slight and that of the lateral CP was large posteriorly in the flexion phase. In the present study, the medial CP translated 3.4 mm posteriorly, and the lateral CP translated 14.2 mm posteriorly with a knee flexion angle from 100° to maxflex. That is, the present results were similar to those results reported previously regarding the flexion phase. On the other hand, in the extension phase, the medial CP translated 8.6 mm anteriorly and the lateral CP translated 9.8 mm anteriorly with a knee flexion angle from maxflex to 100°. There has been no previous report of the translation of CPs in the extension phase. That is, the amount of AP translation of the medial CP was large, as was that of the lateral CP in the extension phase of kneeling. This discrepancy between the flexion and extension phases in the amount of AP translation of the CPs, which is a new finding, could affect the intra-articular components, such as the menisci and cartilage.

The following two points were considered to be the reasons for the kinematic difference between the flexion phase and the extension phase. The first point was the balance of muscle contraction within the hamstrings. Kwak et al. reported that the hamstrings are largely involved in knee joint stability [41]. MacWilliams et al. reported that hamstring co-contraction decreases tibial internal rotation during knee flexion under weight-bearing [42]. The hamstrings produce an efferent contraction during knee extension, and muscle contraction is expected to occur more strongly than during knee flexion. Therefore, the amount of IE rotation was likely reduced in the extension phase compared to the flexion phase, which may have led to this smaller IE rotation in the extension phase. Victor et al. stated that the contraction of the lateral hamstring was responsible for the rotation, particularly the decrease of tibial internal rotation [43]. From these facts, it was thought that the contraction of the lateral hamstring was greatly involved as the cause of the smaller rotation in the extension phase compared to the flexion phase. Second, the sliding-down force of the femur on the tibial plateau surface that was perpendicular to the floor might have affected the knee kinematics during the kneeling activity. At the time of flexion, the femur is already in the position closest to the floor in the starting posture, and deep flexion motion occurs from there. However, when extending, in addition to the anterior translation of the CPs, a force that slides anteriorly along the tibial plateau surface acting in the same direction is added. For these reasons, a phenomenon likely occurs in which the medial CP translates more anteriorly in the extension phase, as in the present results. Therefore, the amount of femoral IE rotation probably differed between the flexion phase and the extension phase in the present study due to the effects of these muscle actions and the force of the femur sliding anteriorly along the tibial plateau surface in the extension phase.

The following points can be considered limitations of this study. First, it was CT-based and did not consider cartilage or meniscal conditions. Therefore, there was a possibility that the point where the cartilage of the femur and the tibia actually contacted each other in vivo and the CP calculated as the geometric centre of the contact area in this analysis did not match completely. Second, kneeling action is usually performed with both legs, but it was performed with one leg in the present study. A handrail was used to perform the kneeling action, so that half of the body weight was applied to the examined knee, as is the case with usual kneeling with both legs. However, it is unclear how much load was actually applied and how much muscle tone was involved. Third, only one sequence, the flexion phase first, followed by the extension phase, was analysed. The analysed knee flexion angles ranged from 100° to maximum flexion in this study. Thus, it is impossible to analyse the kinematics and CPs for the data including angles less than 100°, or in which the order between the flexion phase and the extension phase is reversed. Fourth, electromyography and ground reaction force plate measurements were not performed. Therefore, it is not possible to directly prove the effect of muscle contraction and joint contact pressure in the present kinematic and CP analyses. In the future, it will be necessary to develop methods such as evaluating muscle activity and/or ground reaction force at the same time and standardise the time axis during image capture.

## Conclusions

There was a difference in the IE rotational kinematics between the flexion phase and the extension phase in kneeling. Furthermore, a large amount of anterior translation of the medial CP and a small amount of anterior translation of the lateral CP occurred in the extension phase. These results suggest that the kinematic difference between the flexion and extension phases may have some effects on the meniscus and articular cartilage.

## Data Availability

The datasets used and/or analysed during the current study are available from the corresponding author on reasonable request.

## Abbreviations

SD: Standard deviation
CP: Contact point
OA: Osteoarthritis
AP: Anterior–posterior
IE: Internal–external
IS: Inferior-superior
VV: Varus-valgus
ML: Medial–lateral
CT: Computed tomography
CI: Confidence interval
maxflex: Maximum flexion
